# Fabrication and Characterization of Polycaprolactone–Baghdadite Nanofibers by Electrospinning Method for Tissue Engineering Applications

**DOI:** 10.3390/ma17174187

**Published:** 2024-08-23

**Authors:** Mir Reza Forogh, Rahmatollah Emadi, Mehdi Ahmadian, Abdollah Saboori

**Affiliations:** 1 Department of Materials Engineering, Isfahan University of Technology, Isfahan 8415683111, Iran; mirrezaforogh@gmail.com (M.R.F.); ahmadian@iut.ac.ir (M.A.); 2 Integrated Additive Manufacturing Center, Department of Management and Production Engineering, Politecnico di Torino, Corso Duca degli Abruzzi 24, 10129 Tornio, Italy

**Keywords:** PCL–Baghdadite, electrospinning, bioactivity, degradability, tissue engineering

## Abstract

This work investigates the essential constituents, production methods, and properties of polycaprolactone (PCL) and Baghdadite fibrous scaffolds. In this research, electrospinning was used to produce fiber ropes. In this study, the Baghdadite powder was synthesized using the sol–gel method and incorporated into PCL’s polymeric matrix in formic acid and acetic acid solvents. The present work examined PCL–Baghdadite fibrous scaffolds at 1%, 3%, and 5 wt% for morphology, fiber diameter size, hydrophilicity, porosity, mechanical properties, degradability, and bioactivity. The introduction of Baghdadite nanopowder into pure PCL scaffolds reduced fiber diameter. The wetting angle decreased when Baghdadite nanopowder was added to fibrous scaffolds. Pure PCL reduced the wetting angle from 93.20° to 70.53°. Fibrous PCL scaffolds with Baghdadite nanopowder have better mechanical characteristics. The tensile strength of pure PCL fibers was determined at 2.08 ± 0.2 MPa, which was enhanced by up to 3 wt% by adding Baghdadite nanopowder. Fiber elasticity increased with tensile strength. Baghdadite at a 5% weight percentage reduced failure strain percentage. Fibers with more Baghdadite nanopowder biodegrade faster. Adding Baghdadite ceramic nanoparticles resulted in increased bioactivity and caused scaffolds to generate hydroxyapatite. The results show that Baghdadite PCL-3 wt% fibers have promising shape, diameter, and mechanical qualities. After 24 h, L-929 fibroblast cell viability was greater in the scaffold with 3% Baghdadite weight compared to the pure PCL. PCL-3 wt% Baghdadite fibers generated hydroxyapatite on the surface and degraded well. Based on the above findings, PCL fibers having 3 wt% of Baghdadite are the best sample for tissue engineering applications that heal flaws.

## 1. Introduction

Tissue engineering is a distinct area of study that employs the principles of engineering and biological sciences to fabricate essential tissues to remedy diseases and restore damaged tissues. The discipline above employs cell proliferation, cell culture, tissue matrix construction, and advanced technologies to generate novel tissues. Tissue engineering is a field of study that encompasses two fundamental disciplines, namely bioengineering and histology. Bioengineering is a field of study where engineers apply engineering principles to enhance the diagnosis and treatment of diseases in humans and animals. Histology is a discipline that centers on the characterization, recognition, and comprehension of the role of biological tissues in humans and animals. Tissue engineering is a multidisciplinary field that involves the application of principles from engineering, biology, and medicine to develop functional substitutes for damaged or diseased tissues. Tissue engineering has been identified as a viable approach to address various medical conditions, including skin wounds, vision, and nerve tissues [1,2]. There exist various challenges within the realm of tissue engineering that warrant further investigation in order to address the issues that currently hinder progress in this field. A primary obstacle encountered in tissue engineering pertains to the proper cultivation of cells. In order to generate novel tissues, the body must generate cells in a form that is both of high quality and functionally viable. Notwithstanding, the issues encompass inadequate productivity, weakened cellular robustness, and elevated expenses associated with cultivation.

Furthermore, producing textures with a particular shape poses various difficulties and challenges. The production of novel tissues necessitates the utilization of materials that are both safe and biocompatible. Additionally, other characteristics should be considered depending on their intended application [3,4,5].

In contemporary times, nanofibers in dental applications have garnered significant attention within the scientific and technological communities. Numerous research studies are conducted annually worldwide on this subject matter. It is anticipated that advancements in technology and research will lead to the development of biocomposite materials that exhibit enhanced efficacy and reduced risk. Composite fibers function as a reinforcement phase in response to external forces being applied to the composite material. The force is transmitted to the fibers and subsequently conveyed by them. The utilization of composite fibers in diverse fields and applications, such as mobile prostheses, fixed dental prostheses, restorative dentistry, periodontology, and orthodontics, has been facilitated by the advancement in the development of composite fibers with novel resin systems, as well as improved comprehension of design principles in the production of fiber-reinforced devices. Additionally, these fibers have been employed to mend fractured porcelain veneers. Conducting a thorough assessment of the performance of composite fiber materials currently available in the market and providing appropriate recommendations for patient selection is a crucial aspect that must be considered in order to achieve favorable outcomes [6,7]. In this research, in order to achieve the above characteristics, polymer-based nanofibers with ceramic particles with biocompatibility and biodegradability abilities were selected. Polycaprolactone was chosen as the background phase and Baghdadite as the reinforcing phase [7].

Electrospinning and condensation from the vapor phase are two prominent techniques utilized for commercially producing nanofibers. The prioritization of these methods is based on their higher level of feasibility in comparison to other diverse methods that have yet to attain the desired commercialization point, despite making significant progress. Electrospun and tubular nanofibers are two primary classifications of functional nanofibers that possess a wide range of potential applications in various fields, including but not limited to strength, insulation, electrical conductivity, filtration, biocompatibility, safety, and medical purposes. The electrospinning method is known for achieving precise control over fiber shape and a wide range of diversity in both the raw material and final shape. Nanotube nanofibers produced through this method exhibit exceptional mechanical, thermal, and electrical properties at a low cost [8,9].

The mechanical properties of PCL are advantageous, and it exhibits a prolonged degradation period. PCL exhibits a significant deficiency in bioactivity, and its degradation rate is notably protracted, representing a crucial drawback. Using PCL as a substrate and bioactive ceramic nanoparticles, such as Baghdadite, as a strengthening agent can address the polymer’s limitations [10,11]. Calcium silicate ceramics possessing favorable characteristics such as remarkable bioactivity, commendable mechanical properties, apatite-forming ability, and suitable cell responses are deemed appropriate for employment in tissue engineering [10,12]. Adding elements such as magnesium, zinc, titanium, and zirconium to calcium silicates has been found to enhance their properties as ceramic materials. Baghdadite, an elemental calcium silicate ceramic, has been synthesized using sol–gel techniques and mechanical alloying [13]. They incorporated zirconium ions into the calcium silicate ceramic matrix, resulting in the generation of Baghdadite. Zirconium is a material that exhibits good mechanical strength and biocompatibility, rendering it a popular choice in the field of tissue engineering for repair and reconstruction [14,15]. Baghdadite is a bioceramic material that exhibits biocompatibility and can integrate with the surrounding tissue. Its bioactive properties can potentially expedite the repair process and hasten the degradation of PCL [16,17]. 

The electrospinning of polycaprolactone (PCL) has gained significant attention in biomedical research and regenerative medicine due to its unique properties and versatility. PCL nanofibers fabricated through electrospinning exhibit excellent mechanical properties, biodegradability, and biocompatibility, making them ideal for various applications [18]. Researchers have explored PCL-based electrospun scaffolds for tissue engineering, wound dressing, and drug delivery systems [19,20]. The process parameters, such as applied voltage, flow rate, and polymer concentration, can be optimized to control fiber morphology and diameter [18]. Studies have also focused on creating PCL composites by incorporating other materials like collagen to enhance biological performance [20]. Additionally, surface modifications, such as alkaline hydrolysis, have been investigated to improve the hydrophilicity and functionality of PCL nanofibers for specific applications like lateral flow assays [21,22]. The ongoing research in this field continues to expand the potential of electrospun PCL nanofibers in biomedical applications.

This study involved synthesizing and evaluating the initial Baghdadite nanopowder utilizing the sol–gel technique. PCL fibers were produced and optimized through weight concentrations of 10%, 12%, and 15 wt% following the synthesis of Baghdadite. The production of fibers was carried out under controlled voltage and distance conditions. Subsequently, the impact of incorporating Baghdadite nanoparticles at concentrations of 1, 3, and 5 wt% into a 15 wt% PCL polymer solution was investigated for alterations in mechanical, chemical, and biological characterization properties.

## 2. Materials and Methods

### 2.1. Synthesis of Baghdadite Nanopowder by Sol–Gel Method

Initially, a solution comprising ethanol (C_2_H_6_O, 99%, MERCK, Darmstadt, Germany), Teos (Si(OC_2_H_5_)_4_, 99%, MERCK), and nitric acid (HNO_3_, 65%, MERCK) in a proportion of 8:3:0.16 was prepared and subjected to magnetic stirring at an ambient temperature for 30 min. Subsequently, zirconium nitrate (Zr(NO_3_), 99%, MERCK) was introduced and agitated for 10 min. Following this, calcium nitrate (Ca(NO_3_)_2_·4H_2_O, 99%, MERCK) was incorporated into the solution. The stoichiometric ratio between zirconium nitrate and calcium nitrate is 1:3 [13]. Subsequently, the solution was subjected to magnetic stirring for 5 h, forming a gel. In order to eliminate the residual ethanol, the gel was subjected to thermal treatment by placing it in an oven (MLW WSU100, Labortechnik, Ilmenau, Germany) at 60 °C for one day, followed by exposure to a temperature of 100 °C for two days. Subsequently, calcination was performed by placing the gel in an oven at 1150 °C for three hours. The formed powder was subjected to crushing by being loaded into a zirconia cup and processed in a ball mill for 2 h.

### 2.2. Fabrication Mechanism of PCL Fibrous Nano Scaffolds

**Figure 1:**
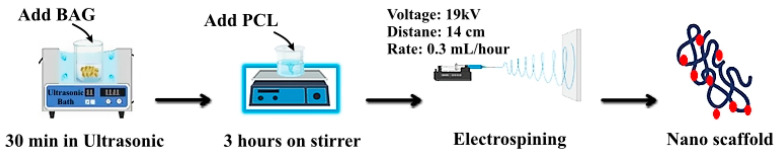
Schematic of polycaprolactone–Baghdadite nano scaffold electrospinning process.

PCL nano scaffolds were produced utilizing 10%, 12%, and 15 wt% weight concentrations. The formation of nano scaffolds was achieved under specific conditions, namely a voltage of 18 kV and a working distance of 16 cm [23]. The PCL polymer was dissolved using a solution of formic acid and acetic acid in a volume ratio of 7:3 and then the polymer was added to the solvent solution. The resultant mixture was subjected to magnetic stirring for 3 h with a magnet. The formed solutions with different weight percentages were poured into 1 mL syringes with a fixed opening of 23 mm and an outer diameter of 0.6 mm. The electrospinning procedure (Medifusion MS-2200, Renton, WA, USA) was conducted at a flow rate of 0.3 mL/h, and the resultant scaffolds accumulated on the aluminum substrate (Figure 1).

### 2.3. Fabrication Mechanism of PCL–Baghdadite Nanocomposite Fibrous Nano Scaffolds

Baghdadite nanoparticles were incorporated into a pure PCL polymer solution using an ultrasonic bath (Delta D-68, Diadema, Brazil) at room temperature for 30 min. The concentrations of the nanoparticles used were 1%, 3%, and 5 wt% by weight to ensure proper and uniform distribution. The pure PCL nano scaffold was subjected to electrospinning for 12 h to disperse better and become uniform while placed on a magnetic stirrer.

### 2.4. Evaluation of Baghdadite Nanopowder

The Baghdadite nanopowder was subjected to an X-ray diffraction (XRD, Philips X-Pert-MPD system, Amsterdam, The Netherlands) analysis. A CuKα lamp with a wavelength of λ = 1.542 A was used to determine the phases formed at 70 > 2θ > 20 and a step size of 0.05.

### 2.5. Analysis of Physical and Chemical Properties of Fibrous Nano Scaffolds

A scanning electron microscope (SEM, Philips XL30) was used to examine the morphology, quantify the fiber diameter, and ascertain the porosity and proportion of surface porosity in distinct fibrous nano scaffolds. In order to examine the morphology of nano scaffolds, the fibrous mesh produced via the electrospinning technique was initially sectioned into 1 cm × 1 cm dimensions and subsequently subjected to a gold coating process utilizing a 10 mA current. Subsequently, three images were captured from distinct locations of each specimen at a voltage range of 10–15 kV. The fiber diameter and porosity of the samples were acquired using ImageJ software, along with the corresponding standard deviation.

The present study aimed to investigate the impact of a high weight percentage of Baghdadite on the hydrophilicity of pure PCL and PCL–Baghdadite nano scaffolds. The contact angle of various samples with water was assessed following the ASTM D5946 standard for analysis [24]. Triads of samples measuring 1 × 1 cm^2^ were fabricated from every composition to achieve this objective. A droplet of water was deposited onto the surface of the specimens, and the contact angle between the droplet and the surface was measured at a time interval of 10 s.

The study employed Fourier-transform infrared spectroscopy (Bomem MB100, Québec, QC, Canada) to examine and explore the bonding mechanisms in fibrous nano scaffolds. The spectral analysis was conducted within the 4000–400 cm^−1^ range, with a scanning rate of 2 cm^−1^.

To confirm the presence of Baghdadite nanoparticles within the nanofibers, X-ray diffraction analysis was conducted using a CuKα lamp with a wavelength of λ = 1.542 at an angle of 20 > 2θ > 70 and a step size of 0.05. The X-ray diffraction patterns were analyzed using X-Pert software Version 3.0d and reference data from standard cards.

### 2.6. Analysis of Mechanical Properties of Nano Scaffolds

A tensile test was conducted to evaluate the mechanical characteristics of the nanofibers that were produced. The conducted test was uniaxial and was executed following the established ASTM D3822 protocol with a tensile test machine (Hounsfield H25KS) [25]. Fibrous nano scaffolds were used to prepare samples with 10 × 70 mm^2^ dimensions to facilitate testing. Stress–strain curves were generated for various specimens utilizing an Instron apparatus with a 5 kg load cell. The specimens were subjected to a tensile velocity of 10 mm/min and a 10 N load. The experiment was conducted with triplicate trials for each sample, and the failure strain and tensile strength of the specimens were evaluated by plotting the data of various samples alongside the corresponding standard deviation.

### 2.7. Investigating the Biodegradability of Fibrous Nano Scaffolds

Subsequently, a phosphate buffer solution was added to individual falcon tubes at a temperature of 37 degrees Celsius in a quantity equivalent to 100 times the mass of each respective sample. The falcons were subjected to a controlled environment in a Bain-Marie (EYELA T-80) apparatus, maintained at a constant temperature of 37 °C for four weeks. The samples were retrieved at 7, 14, 21, and 28 days weekly. Following a rinse with distilled water, the samples were desiccated to achieve complete drying and weighed to obtain their respective mass (M). The biodegradable solution was retrieved at regular intervals of three days to ensure consistency. The weight reduction percentage was determined per the ASTM D570 standard utilizing a formula [26]. The formula for mass loss can be expressed as a percentage using the following equation: Mass Loss = [(M − M0)/M0] × 100.

### 2.8. Bioactivity Analysis of Fibrous Nano Scaffolds

The bioactivity test commenced by preparing the simulated body solution following the guidelines [27]. For the bioactivity assay, nano scaffolds were partitioned into 10 × 10 mm^2^ dimensions, and 10 mL of the prepared solution was introduced into each falcon along with the sample. The falcons were then subjected to a Bain-Marie bath at a temperature of 37 degrees Celsius for four weeks. The subjects underwent weekly intervals of bath removal, specifically on the 7th, 14th, 21st, and 28th days. A scanning electron microscope was employed to examine the bioactivity and morphology of apatites on nano scaffolds. X-ray energy dispersive microanalysis (EDX) examined the proportion of apatite constituent elements. The concentration variations of phosphorus and calcium ions were examined using an atomic emission spectrometer, specifically an inductively coupled plasma (ICP) test (AES Varian).

### 2.9. Cells Culture

The MTT assay (3-(4,5-dimethylthiazol-2-yl)-2-5-diphenyltetrazolium bromide) is used (along with 10% fetal bovine serum (FBS), 1% penicillin, and streptomycin) (Nano Ala, Isfahan, Iran) to evaluate cell viability in response to the nanoparticle samples, providing an assessment of the samples’ biocompatibility. In live cells, the MTT dye is reduced in the mitochondria, changing from a yellow color to purple formazan crystals. The concentration of this color is an indicator of the number of viable cells, which can be measured using a photometer. To evaluate and compare polycaprolactone scaffolds and optimized scaffolds containing Baghdadite nanoparticles, they were exposed to cell interaction. This test and its evaluation criteria are conducted according to ISO 10993-5:2009 standards [28]. The equipment used in this assay includes a laminar hood, an incubator (37 °C, 5% CO_2_), a centrifuge, an ELISA reader, and an inverted microscope.

## 3. Results and Discussion

### 3.1. Preparation of Baghdadite Nanopowder

**Figure 2:**
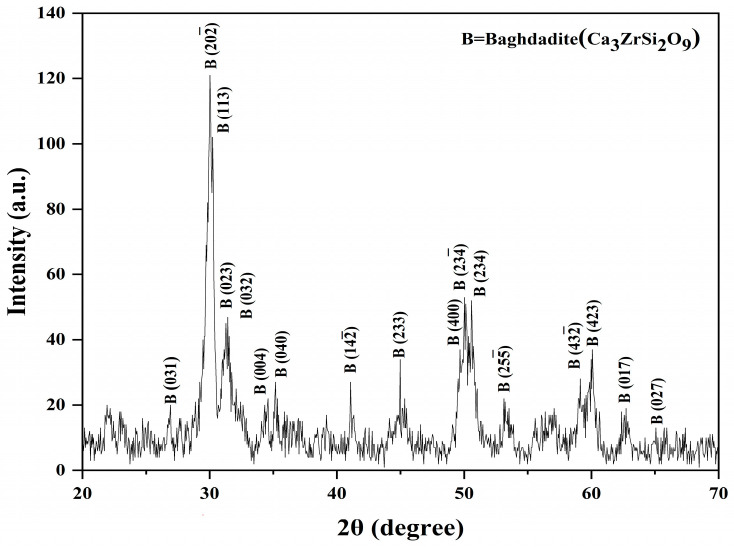
X-ray diffraction pattern of Baghdadite nanopowder.

The sol–gel technique was employed to synthesize Baghdadite nanopowder, which was subsequently subjected to ball milling for 2 h in a zirconia cup rotating at a velocity of 250 revolutions per minute. Figure 2 displays the X-ray diffraction pattern of the powder that was produced. The diffraction pattern obtained was analyzed using X-Pert software. The peaks observed were compared to the established standard of Baghdadite, identified by the code JCDP: 00-016-0155. The phases of Baghdadite were determined based on the intensity of the peaks and their diffraction angles. The confirmation of the Baghdadite phase was achieved through an examination of both the formed phases and the established standard.

### 3.2. Fabrication of PCL Fibrous Scaffold

**Figure 3:**
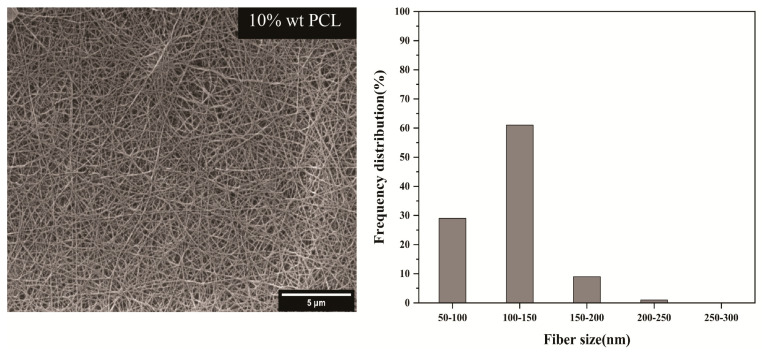

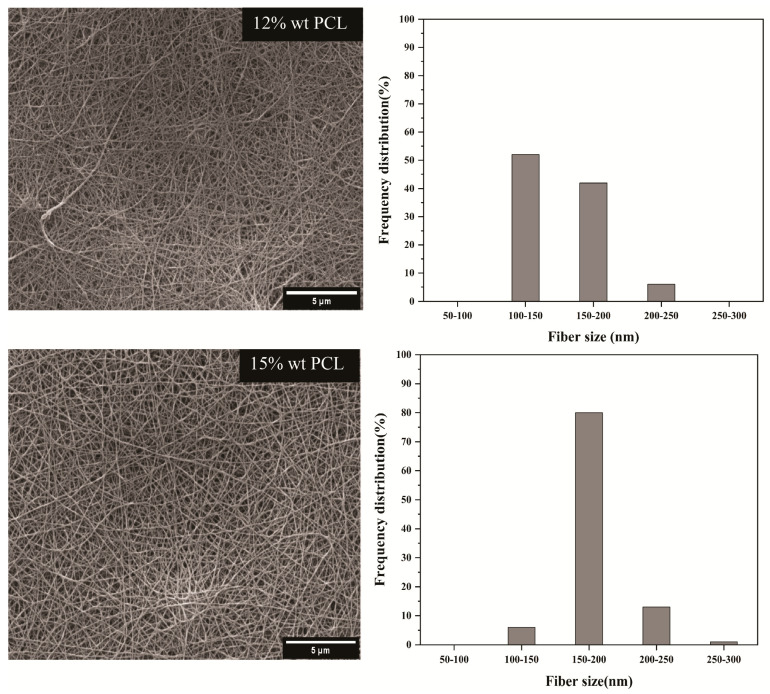
SEM images and nanofiber diameter dimension distributions histograms.

The present study employed acetic acid and formic acid as solvents for PCL. Both solvents exhibit low toxicity, and formic acid is utilized singularly in electrospinning the amalgamation of formic acid and acetic acid. The substance was formulated in a proportion of 7:3 and has demonstrated favorable efficacy as a replacement for chloroform [29,30,31]. The optimization of the solution concentration was investigated by utilizing varying weight percentages of PCL, specifically 10%, 12%, and 15 wt%. In Figure 3, fibers with a concentration of 10 wt% PCL are observed in drop fibers due to the low viscosity and tensile strength of fibers and a non-uniform structure. The findings in Figure 3 indicate that the presence of 12 wt% PCL in willow fibers reduces their mechanical properties and tensile strength. The fibers comprising 15 wt% PCL exhibit uniform strands devoid of visible willows, as depicted in Figure 3. Furthermore, through augmentation of the quantity of PCL, a corresponding increase in the diameter of the fibers was observed. The diameter of the fibers in all three percent by weight was in the range of 100–200 nm. In the amount of 15 wt%, the formed fibers had fewer knots in addition to the uniform structure and without willow. Also, the diameter of the fibers had a minor deviation from the standard than the other weight percentages of PCL. With the interpretations and investigations, the concentration of 15 wt% PCL was chosen as the optimal concentration.

### 3.3. Fabrication and Physical and Chemical Properties of the PCL–Baghdadite Scaffold

**Figure 4:**
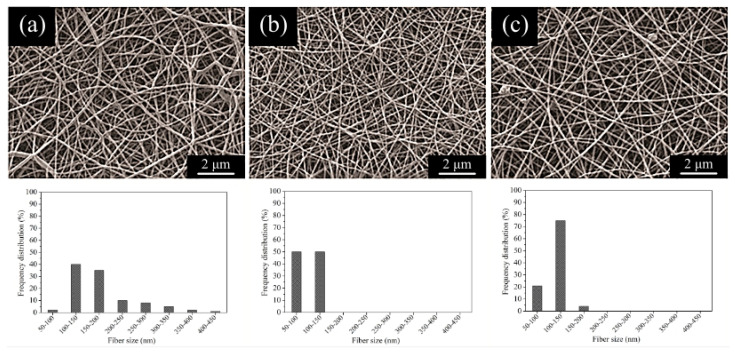
SEM images and nanofiber diameter dimension distribution histograms of (**a**) PCL-1 wt% BAG, (**b**) PCL-3 wt% BAG, and (**c**) PCL-5 wt% BAG fibrous scaffolds.

As reported in a reference, the electrospinning procedure involves decreasing the solution’s viscosity and enhancing its conductivity to produce fibers with reduced diameter [32]. As previously noted, Kharaziha and his associates utilized forsterite ceramic particles to decrease viscosity and enhance conductivity, thereby reducing fiber thickness [33]. Previous research has indicated that the inclusion of ceramic particles such as silica [34] and hydroxyapatite [35] in PCL polymer fibers has reduced the diameter of said fibers. The micrographs presented in Figure 4 depict the surface of the fabricated fibers, as captured by a scanning electron microscope. The figure showcases several distribution diagrams, which reveal that the incorporation of Baghdadite nanopowder results in a reduction in fiber diameter as the weight percentage of the nanopowder increases. The findings in Table 1 illustrate a negative correlation between the size of ceramic particles and fiber diameter. Incorporating Baghdadite nanopowder at 1 wt% does not yield a statistically significant alteration in the mean fiber diameter (Figure 4a). Fibers that incorporate 3 wt% Baghdadite nanopowder exhibit a noticeable reduction in fiber diameter, with the lowest observed fiber diameter (Figure 4b). Baghdadite nanopowder at a weight percentage of 5 wt% in fibers results in agglomeration within the fibers, which is attributed to the heightened concentration of ceramic particles (Figure 4c). The observed increase in the viscosity of the solution has resulted in reduced fiber strength emanating from the stretching jet. Consequently, fibers containing 5 wt% Baghdadite nanopowder exhibit greater thickness compared to those containing 3 wt% of the same nanopowder [10].

Conversely, as the Baghdadite nanopowder concentration increased, the fibers’ porosity decreased. This resulted in a reduction in both the thickness and diameter of the fibers. The decrease in fiber size led to an increase in the number of layers that could accumulate on top of one another. Furthermore, it has been observed that there is a reduction in porosity as a consequence [36]. Baghdadite fibers exhibit the most negligible thickness, and consequently, the porosity is minimized in PCL with a weight percentage of 3 wt%. Baghdadite at a weight percentage of 5 wt% in the fibers is associated with a slight increase in porosity as thickness increases.

**Figure 5:**
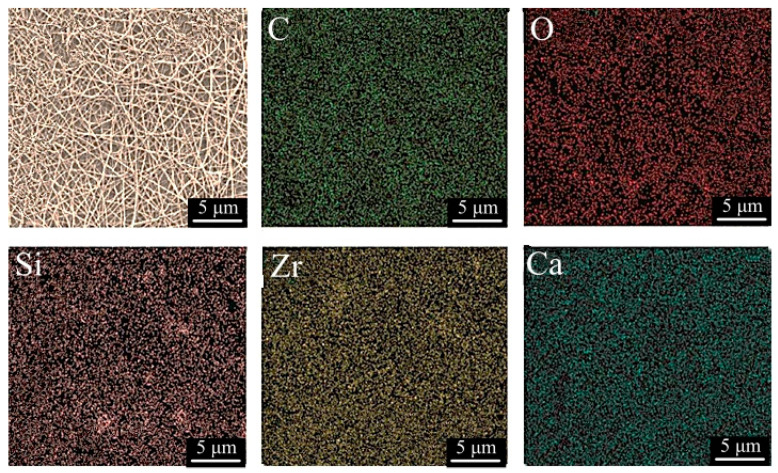
Elemental analysis of electrospun nanocomposite scaffold of PCL-3 wt% BAG.

The distribution of the elements in the PCL-3 wt% Baghdadite scaffold is shown in Figure 5 using elemental color map analysis. According to the shape and distribution of the elements, the uniform presence of Baghdadite ceramic nanoparticles in the polymer field is confirmed. The elemental distribution of carbon, oxygen, silicon, zirconium, and calcium within the fibers is illustrated.

**Figure 6:**
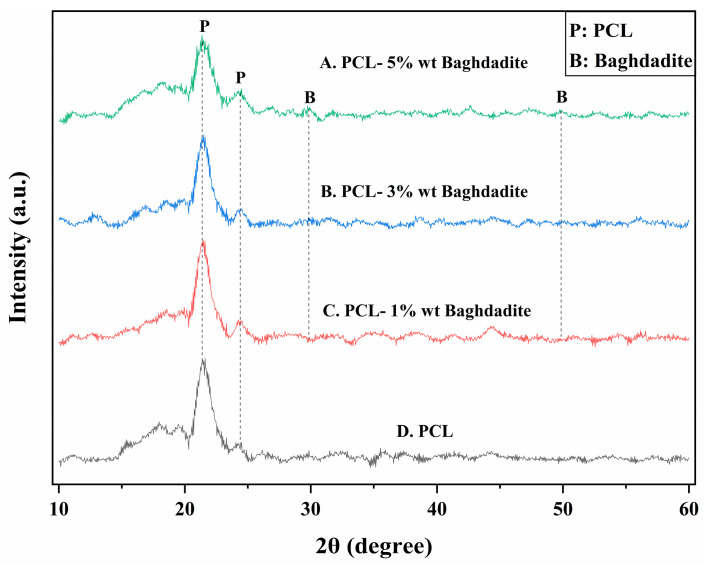
X-ray diffraction pattern of PCL–Baghdadite scaffold composites.

The scaffolds were subjected to X-ray diffraction analysis, as depicted in Figure 6, to obtain their respective diffraction patterns. The presence of Baghdadite nanopowder within PCL fibers can be substantiated based on observed patterns. The peaks observed at 2θ = 29.75 and 2θ = 49.9 degrees are attributed to Baghdadite. It was observed that the intensity of these peaks marginally increased with the rise in the weight percentage of Baghdadite. Based on the X-ray diffraction pattern analysis of the pure polymer sample, it can be inferred that the two distinct peaks observed at angles 2θ = 21.4 and 2θ = 24.35 degrees correspond to PCL. The addition of Baghdadite nanopowder reduced the peak intensity, indicating an increase in the weight amount. Based on the study’s findings, it can be inferred that incorporating Baghdadite nanoparticles into PCL resulted in a composite material. The observed reduction in polymer peaks and increase in Baghdadite nanopowder peaks supports this conclusion and is deemed valid [33,37].

**Figure 7:**
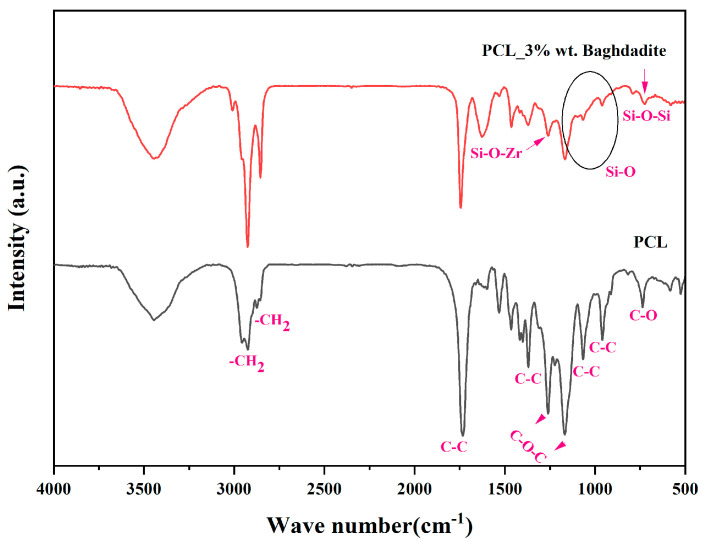
Fourier transforms of the infrared spectrum of pure PCL scaffold and PCL-3 wt% Baghdadite.

A Fourier-transform infrared spectrometer analysis was conducted on a scaffold made of pure PCL and a composite scaffold consisting of PCL and 3 wt% Baghdadite. The peaks acquired have been depicted in Figure 7. The length of a peak in a chemical structure indicates the percentage of the bond associated with that peak. Additionally, a sharper peak indicates a greater degree of order in the substance’s chemical structure. This spectroscopic technique involves the analysis of peak shifts, removal, creation, and modulation of peaks during material processes to investigate structural modifications and substitutions. The present study depicts the display of links in diverse wavelengths based on the analysis and interpretation of the peaks derived from the polymer scaffold and the PCL-3 wt% Baghdadite scaffold [37,38,39].

**Figure 8:**
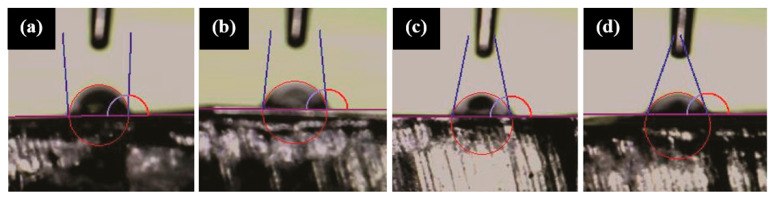
Images of wettability test: (**a**) PCL, (**b**) PCL-1 wt% BAG, (**c**) PCL-3 wt% BAG, and (**d**) PCL-5 wt% BAG fibrous scaffolds.

A decrease in wetting angle was observed with an increase in the weight percentage of Baghdadite. The pure PCL scaffold exhibited a wetting angle of 93.2 ± 2 degrees (Figure 8a). Subsequently, the scaffolds that comprised PCL–Baghdadite were subjected to varying weight percentages of Baghdadite, namely 1%, 3%, and 5%. As a result, measurements of 84.56 ± 1, 76.93 ± 2.2, and 70.53 ± 1.2 degrees were acquired (Figure 8b–d). In a comparable investigation, ref. [23] assessed wettability. The study demonstrated that augmenting the weight of diopside nanopowder by 20% in a silk polymer scaffold reduced the wetting angle from 86.41 ± 3 degrees to 76.98 ± 5 degrees. The authors found that the primary factor contributing to the reduction in the wetting angle is the escalation in the surface energy of the scaffold, which is directly proportional to the augmentation of ceramic nanoparticles. The contact between the water droplet and the surface of the fibrous scaffolds and the resulting angle is depicted in Figure 8.

### 3.4. Mechanical Properties of Fibrous Scaffolds

**Figure 9:**
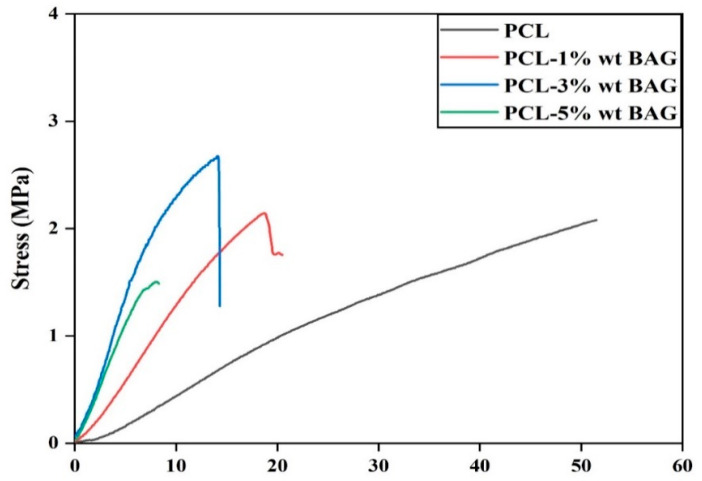
Stress–strain diagram of fibrous scaffolds containing different percentages of Baghdadite nanopowder.

The mechanical properties of fiber scaffolds are a crucial and contentious topic. The stress–strain diagram of the fabricated fiber scaffolds is depicted in Figure 9. The specimens initially underwent elastic deformation and transitioned into the plastic deformation regime, culminating in failure upon reaching the yield point. Table 2 presents the tensile strength, breaking strain, and elastic modulus values for various composite scaffolds.

The tensile strength of the PCL fibrous scaffold was observed to reach its maximum value upon the addition of 3 wt% Baghdadite nanopowder, resulting in a value of 2.08 ± 0.2 MPa. Upon increasing the weight percentage of Baghdadite nanopowder to 3 wt%, the value above one was observed to reach 2.67 ± 0.1 MPa. The observed enhancement in strength can be attributed to the reduction in fiber diameter resulting from incorporating Baghdadite nanoparticles [32]. The augmentation in strength can be attributed to the energy absorption of fibers in the existence of ceramic nanoparticles. Under tensile force, the ceramic particles assimilate this force and generate a provisional network amidst polymer chains and ceramic nanoparticles [40]. The augmentation of the weight percentage of Baghdadite nanopowder resulted in a reduction in strength to 5 wt% owing to agglomeration, which refers to the aggregation of nanoparticles and non-uniformity in the distribution of fibers. In a separate investigation, it was observed that the fibrous scaffold composed of PCL–forsterite exhibited superior and more favorable characteristics compared to the scaffold made solely of PCL. Incorporating more significant quantities of forsterite resulted in a 20% reduction in mechanical properties by mass [33]. In a comparative investigation, it was observed that the incorporation of 3 wt% diopside nanopowder into PCL–diopside fibers resulted in improved mechanical properties in comparison to pure PCL fibers, as well as PCL fibers containing 5% and 7% by weight of diopside nanopowder [36]. The present study investigated the effect of the weight percentage of Baghdadite nanopowder on the failure strain of PCL–Baghdadite composite fibers. The findings revealed that an increase in the weight percentage of Baghdadite nanopowder resulted in a decrease in the failure strain. This can be attributed to the increased hardness and decreased flexibility of the composite fibers with the increase in Bagdadite nanopowder content. Based on the elastic modulus values and the underlying mechanism for enhancing mechanical properties, it can be posited that the scaffold incorporating 3 wt% Baghdadite exhibits superior strength, increased rigidity, and reduced flexibility. The optimal sample for mechanical properties was determined to be a PCL scaffold containing 3 wt% Baghdadite nanopowder due to its favorable physical and structural characteristics.

### 3.5. Biodegradability of PCL–Baghdadite Fibrous Scaffolds

**Figure 10:**
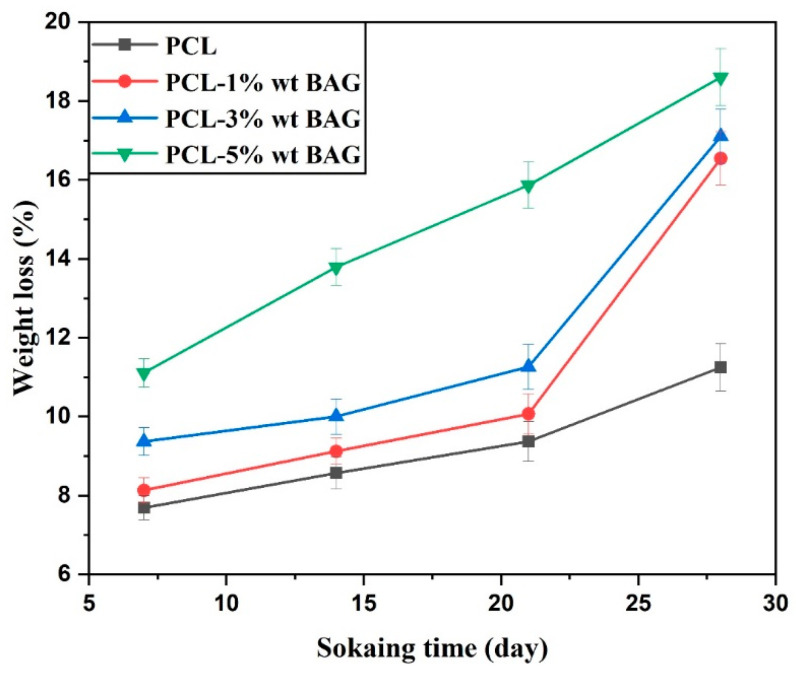
Weight loss percentage diagram of PCL–Baghdadite nanocomposite fibrous scaffolds.

The results of the degradability test conducted on fibrous scaffolds indicate that the incorporation of Baghdadite nanopowder in increasing weight amounts led to an enhancement in the biodegradable characteristics of the scaffolds. As per prior assertions, the fibrous scaffold composed of pure PCL exhibits limited degradability. However, incorporating Baghdadite nanopowder has been demonstrated to enhance this attribute. The graph depicted in Figure 10 illustrates the extent of weight loss and destruction observed in various fibrous scaffolds. Upon completing the degradability assessment, specifically after 28 days, the PCL scaffold exhibited a weight reduction of 11.11 ± 1.2%. Meanwhile, the scaffolds infused with 1, 3, and 5 wt% Baghdadite nanopowder demonstrated weight losses of 13.79 ± 1.6%, 15.87 ± 2.1%, and 18.60 ± 2.5%. Upon immersion of the fibrous scaffolds in a phosphate buffer solution, a gradual weight reduction was observed, with the maximum weight loss occurring after the third week. Upon immersion of the scaffolds in a phosphate buffer solution, the polymer chains comprising the scaffolds undergo separation from one another, facilitated by the ingress of water into the amorphous regions of the polymer. Over time, the polymer’s crystalline region experiences water infiltration, leading to hydrolysis and subsequent weight reduction. The introduction of Baghdadite nanopowder into the fibers dissociates calcium ions within the scaffolds, leading to hydrolysis of the Si-O-Si bonds present on the fiber surface. This process facilitates the formation of new Si-OH bonds. This phenomenon disrupts the structural support connections, resulting in a reduction in body mass. It can be posited that a lack of interaction between the polymer matrix and ceramic nanoparticles results in the facile placement of Si-OH groups between them, leading to increased water penetration, degradation, and weight loss of the scaffolds. Furthermore, it can be posited that the degradation process is heightened by reducing fiber thickness and diameter, owing to ceramic nanoparticles and increased surface roughness [23].

### 3.6. Bioactivity of Nanocomposite Fibrous Scaffolds

**Figure 11:**
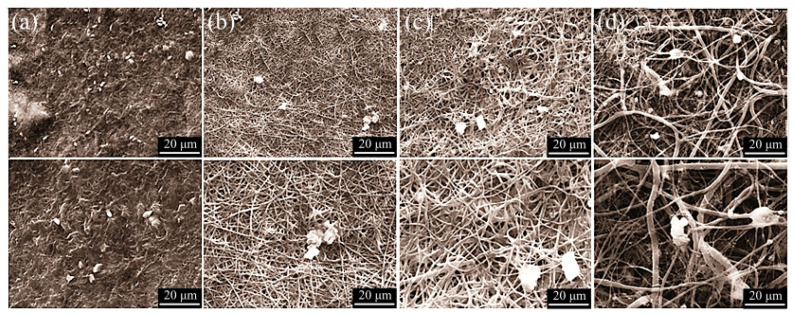
SEM images after 28 days of immersion in the simulated body solution of (**a**) PCL, (**b**) PCL-1 wt% BAG, (**c**) PCL-3 wt% BAG, and (**d**) PCL-5 wt% BAG fibrous scaffolds.

Bioactivity is a crucial characteristic of implants and scaffolds, forming a hydroxyapatite layer at the interface between the scaffold and the biological milieu. According to a scholarly source, hydroxyapatite’s chemical composition and structure resemble that of the mineral phase of bone. The fibrous scaffolds underwent immersion in a simulated body solution, forming apatites on the scaffold’s surface. Prior studies have demonstrated that Baghdadite exhibits favorable bioactivity [41,42]. The results depicted in Figure 11 demonstrate apatite formation through the gradual increase in the weight percentage of Baghdadite nanopowder on the fiber surface following a 28-day immersion period in a body simulator solution. The bone growth potential of the PCL (Figure 11a) scaffold has been observed after 28 days of immersion in SBF solution. However, it does not exhibit any distinct advantage in this regard [33]. Roohani et al. [13] demonstrated that Baghdadite exhibits favorable bioactivity and biocompatibility characteristics and superior mechanical properties compared to alternative calcium phosphate compounds.

The gradual formation of apatites was observed upon the addition of Baghdadite nanopowder. The fibers that comprise 1 wt% Baghdadite exhibit visible primary apatite buds on their surface (Figure 11b). The augmentation of Baghdadite nanopowder to 3 and 5 wt% Baghdadite resulted in a corresponding increase in the quantity of apatites and their growth (Figure 11c,d). Introducing Baghdadite into the fibrous scaffolds modifies the apatite morphology upon immersion in a simulated body solution. Specifically, the needle-shaped physical state of the apatite transforms into a spherical and prismatic form due to the release of silicon ions into the solution and competes with others [43]. Hydroxyapatite is the most stable, dense, and insoluble form of calcium phosphate. The principal mode of bioactivity involves the initiation of apatite germination on the scaffold’s surface through dissolution, sedimentation, and growth. The process begins with the partial dissolution of Baghdadite in the scaffolds in the simulated body solution. This can be attributed to calcium and phosphorus ions in the supersaturated solution, which precipitates ions like calcium and phosphorus. The process occurs from the surrounding environment towards the exterior surface of the scaffolding. Ions undergo movement and rearrangement on the surface of the scaffold. The initial nucleation of hydroxyapatite occurs, followed by subsequent ion deposition onto the scaffold surface, leading to the growth of hydroxyapatite crystals over time.

**Figure 12:**
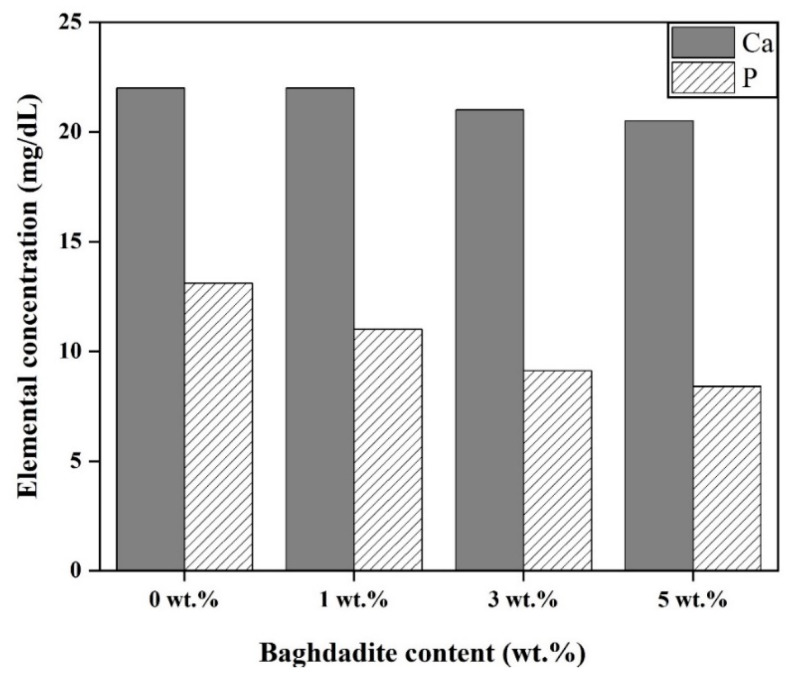
The concentration of Ca and P ions in an SBF solution after 28 days of immersion of PCL–Baghdadite fibrous scaffolds.

The alterations in the levels of calcium and phosphorus ions to the augmentation of the weight percentage of Baghdadite nanopowder in the PCL–Baghdadite scaffolds within the simulated body solution after 28 days are depicted in Figure 12. The release of zirconium from the Baghdadite nanopowder is facilitated by the displacement of Baghdadite nanoparticles and positive ions in the simulated body solution, which is achieved by increasing the weight percentage of the nanopowder. The bioactivity of Baghdadite is induced by the reaction above. The breaking of silica bonds in nanoparticles is attributed to the displacement of ions, leading to the formation of Silanol (Si-OH) bonds on the surface of the scaffolds, which are hydrophilic. The calcium concentration has remained relatively stable due to the liberation of calcium ions from the Baghdadite structure. A marginal reduction has been observed in conjunction with an increase in Baghdadite nanopowder concentration, resulting in a corresponding decrease in the concentration of phosphorus ions within the simulated body solution. The observed alterations substantiate the development of the apatite phase on fibrous scaffolds. In a separate investigation, the augmentation in a weight proportion of diopside nanoparticles resulted in the liberation of calcium ions within the scaffold that encompassed a 3% weight of diopside nanopowder. This led to a surge in the concentration of calcium ions, which was subsequently followed by a decline. The findings suggest a decrease in the concentration of phosphorus ions as the diopside nanopowder increased [44].

**Figure 13:**
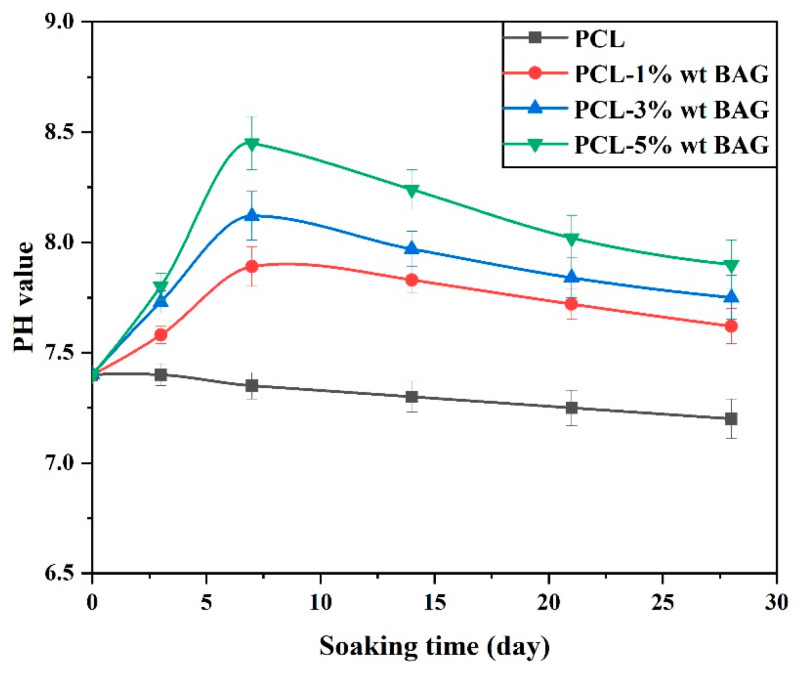
pH values of the simulated body solution along with PCL–Baghdadite fibrous scaffolds.

The pH level of the fibrous scaffolds submerged in the simulated body solution was assessed for 28 days, and the outcomes are depicted in Figure 13. Initially, the liberation of zirconium ions from Baghdadite nanoparticles takes place, forming hydrophilic Silanol groups. The interactions above result in the deposition of calcium and phosphorus ions from the simulated supersaturated solution of the human body onto the Silanol group’s bonds. The pH of the pure PCL scaffold exhibited a slight decrease for 28 days, ultimately reaching a value of 7.2 from an initial value of 7.4. The samples comprising Baghdadite ceramic nanoparticles exhibited a rise in pH during the initial week, followed by a subsequent decline in the next week. After one week, the pH value of the solution in the PCL fibrous scaffold containing 5 wt% Baghdadite reached its maximum value of 8.45. Subsequently, the PCL underwent degradation over a while. The acidity of the environment increased concomitantly with a decrease in pH. The primary cause of the rise in pH of the simulated body solution is attributed to the interaction between zirconium and calcium ions with H+ ions (protons) present in the simulated body solution. In this substitution, the concentration of protons in the solution decreases. The substance has been located, and the pH level is recommended to be elevated. The findings and examination indicate that the incorporation of Baghdadite nanopowder into the PCL polymer scaffold has enhanced the scaffolds’ bioactivity. This can be attributed to the discharge of zirconium ions, which create a favorable environment on the fibrous scaffold surface to develop the apatite phase.

Furthermore, segregating ions and nanoparticles from the scaffold augments surface roughness, thereby generating favorable sites for apatite deposition and germination. This, in turn, facilitates the growth of the apatite phase on the surface of the scaffolds. Based on the phosphorus ion concentration in the simulated body solution after 28 days, it can be concluded that the lower pH value increase observed in solutions with scaffolds containing a lower weight percentage of Baghdadite nanopowder can be attributed to the higher concentration of phosphorus ions in the solution. This phenomenon results in the formation of solution buffering and a reduction in pH.

### 3.7. Evaluation of Biocompatibility in Fibrous Nanocomposite Scaffold Structures

**Figure 14:**
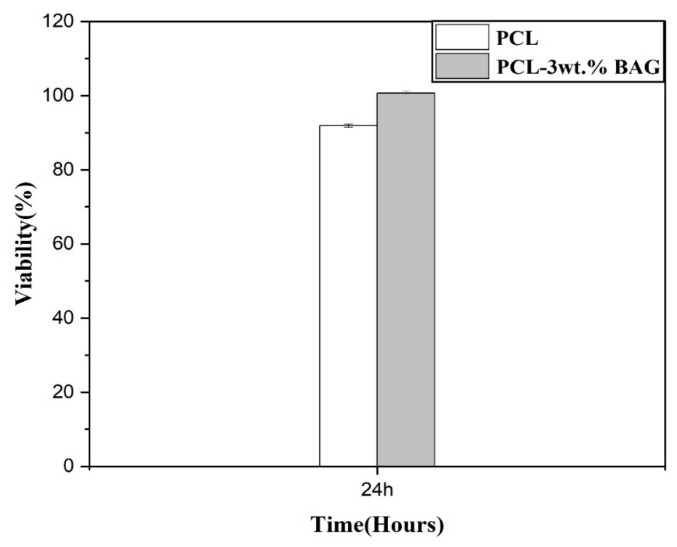
Results of fibroblast cell culture and specimen distribution among cells during 24 ± 2 h period.

In this section, the viability of L-929 fibroblast cells will be addressed. Analyses after 24 ± 2 h revealed that the cell viability in the scaffold containing a 3% weight of Baghdadite is higher compared to the scaffold of pure polycaprolactone, demonstrating greater biocompatibility. Additionally, cell adhesion is found to be lower in the Baghdadite-incorporated scaffold. Consequently, it can be asserted that this scaffold is more suitable for biological applications and exhibits superior properties. The viability levels at 24 ± 2 h are illustrated in Figure 14.

**Figure 15:**
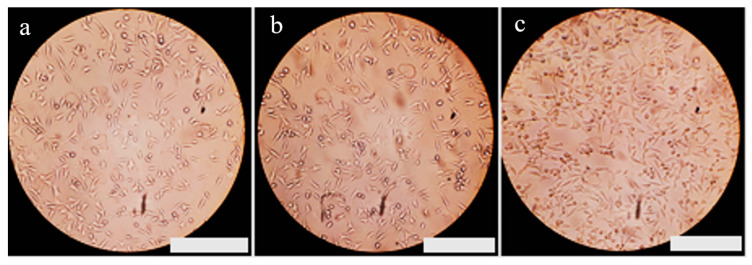
Images of cells in the control sample and exposed to samples during the 24 ± 2 h period. (**a**) Control sample, (**b**) polycaprolactone, and (**c**) polycaprolactone with a 3% weight of Baghdadite. Scale bar: 500 μm.

The cell viability in the polycaprolactone scaffold was approximately 92.91%. However, with the addition of a 3% weight of Baghdadite nanoparticles, the cell viability reached 100.72%. Factors such as surface roughness, hydrophilicity, and the chemical composition of materials directly influence cell growth and proliferation. The morphology of the developed cells on the surface plays a significant role in determining the biocompatibility of implants [45]. Moreover, it was demonstrated that the incorporation of Baghdadite nanoparticles into the scaffold increases its hydrophilicity. Figure 15 presents images obtained from optical microscopy of the samples after 24 ± 2 h of cultivation (Figure 15a: control sample, Figure 15b: polycaprolactone, and Figure 15c: Polycaprolactone with a 3% weight of Baghdadite). These findings suggest a nuanced relationship between Baghdadite content, cell viability, and hydrophilicity in the context of scaffold biocompatibility.

**Figure 16:**
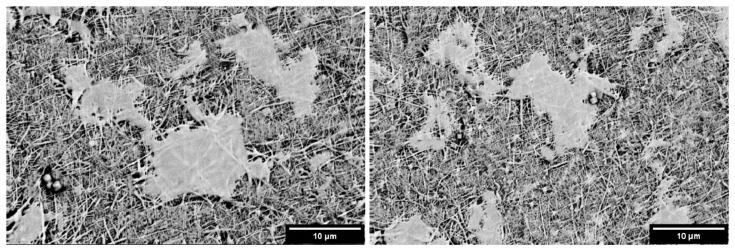
A scanning electron microscopy image of the cell attachment on the polycaprolactone scaffold with a 3% weight of Baghdadite.

Figure 16 depicts cells after 24 ± 2 h of cultivation on scaffolds containing a 3% weight of Baghdadite. The images illustrate that these scaffolds possess the capability for cell growth and exhibit favorable adhesion onto the scaffold surface. The enhancement of cellular performance has been observed in various studies with the incorporation of ceramic nanoparticles, such as diopside into silk fibroin polymer scaffolds [23], the addition of beta-tricalcium phosphate ceramic nanoparticles to polyglycolic acid scaffolds [46], and the incorporation of acemannan nanoparticles into poly-caprolactone scaffolds [47]. These instances highlight the positive impact of ceramic nanoparticles on cellular functionality within diverse polymeric scaffolds.

## 4. Conclusions

The electrospinning technique produced fibrous scaffolds composed of PCL and Baghdadite nanoparticles at varying concentrations. The resulting PCL–Baghdadite nanocomposite fibrous scaffolds were subsequently assessed. The analyses and reviews indicated the following:

- The incorporation of Baghdadite nanopowder into PCL nanofibers resulted in a reduction in the average thickness of the fibers. Notably, the fibers containing 3 wt% Baghdadite nanopowder exhibited the lowest thickness, and a more uniform size distribution was observed in the formed fibers. The introduction of additional Baghdadite nanopowder resulted in agglomeration within the fibers, increasing the mean diameter of said fibers.
- The mechanical characteristics of PCL fibers were enhanced by a 3 wt% increase in Baghdadite nanopowder. This resulted in the scaffold’s elasticity coefficient and tensile strength reaching their maximum levels compared to other fibers. The introduction of additional Baghdadite nanopowder into the scaffolds results in a reduction in mechanical properties. This can be attributed to the emergence of stress concentration sites arising from the agglomeration of ceramic nanoparticles within the field. Furthermore, the lack of nanoparticle mobility along the path of tensile force exacerbates this effect.
- The incorporation of Baghdadite nanopowder into PCL fibers resulted in a reduction in the wetting angle and an increase in the degradation rate of fibrous scaffolds when subjected to immersion in a PBS solution for 28 days.
- The incorporation of Baghdadite nanopowder into PCL resulted in an enhancement of the bioactivity of fibrous scaffolds composed of nanocomposites.
- The cell viability in the scaffold containing a 3% weight of Baghdadite is higher compared to the scaffold of pure polycaprolactone after 24 ± 2 h, and other researchers can perform animal tests and analyze the behavior and response of the scaffolds under physiological conditions.

## Figures and Tables

**Table 1:** The average diameter of fibers and the percentage of surface porosity of composite nanofibers.

Scaffold	Fiber’s Diameter Average (nm)	Porosity (%)
PCL	177.5 ± 23.5	71.74 ± 3.7
PCL-1 wt% BAG	177.1 ± 66.7	69.07 ± 2.1
PCL-3 wt% BAG	100.9 ± 16.4	63.30 ± 1.8
PCL-5 wt% BAG	114.8 ± 17.4	64.54 ± 3.5

**Table 2:** The mechanical properties of PCL–Baghdadite scaffold composites.

Scaffold	Fracture Strength (MPa)	Strain at Fracture (%)	Elastic Modulus (MPa)
PCL	2.08 ± 0.0	51.50 ± 7.8	5.40 ± 0.02
PCL-1 wt% BAG	2.14 ± 0.3	21.35 ± 1.65	13.92 ± 0.02
PCL-3 wt% BAG	2.67 ± 0.1	14.03 ± 2.67	30.57 ± 0.10
PCL-5 wt% BAG	1.5 ± 0.05	8.30 ± 5.1	24.05 ± 0.05

## Data Availability

The original contributions presented in the study are included in the article, further inquiries can be directed to the corresponding authors.
